# Infection-driven proteomic signatures in immune cell–derived extracellular vesicles reflect hemorrhagic stroke outcome

**DOI:** 10.1186/s12974-025-03635-9

**Published:** 2025-12-04

**Authors:** Fernando Laso-García, Elisa Alonso-López, Dolores Piniella, Exuperio Díez-Tejedor, Mari Carmen Gómez-de Frutos, Laura Casado-Fernández, Laura Otero-Ortega, Mari Paz López-Molina, Rebeca Gallego-Ruiz, Javier Pozo-Novoa, Ángela Calzado-González, Nerea Díaz-Gamero, Alicia Román-San Martín, Susana Bravo, Rodrigo Barderas, Félix Docando, Blanca Fuentes, Belén Juárez-Martín, María Alonso de Leciñana, María Gutiérrez-Fernández

**Affiliations:** 1 Neurological Sciences and Cerebrovascular Research Laboratory, Department of Neurology and Stroke Centre, Neurology and Cerebrovascular Disease Group, Neuroscience Area La Paz Institute for Health Research (idiPAZ), (La Paz University Hospital- Universidad Autónoma de Madrid), Madrid, Spain; 2 Translational Stroke Laboratory (TREAT), Clinical Neurosciences Research Laboratory (LINC), Health Research Institute of Santiago de Compostela (IDIS), 15706 Santiago de Compostela, Spain; 3 Faculty of Medicine, Universidad Alfonso X el Sabio, Villanueva de la Cañada, Madrid, Spain; 4 Faculty of Health Sciences - HM Hospitals, University Camilo José Cela, Madrid, España; 5 Instituto de Investigación Sanitaria HM Hospitales, Madrid, 28015 Spain; 6 Proteomic Unit, Health Research Institute of Santiago de Compostela (IDIS), Santiago de Compostela, Spain; 7 Chronic Disease Program, UFIEC, Carlos III Health Institute, Madrid, Spain; 8 CIBER Frailty and Healthy Aging (CIBERFES), Madrid, Spain; 9 Electron Microscopy Unit, Scientific-Technical Central Units, Carlos III Health Institute (ISCIII), Majadahonda, Madrid, 28220 Spain

**Keywords:** Extracellular vesicles, Hemorrhagic stroke, Infection, Proteomic analysis

## Abstract

**Background:**

Analyzing the content of immune cell-derived extracellular vesicles (EVs) may reveal biomarkers that elucidate the mechanisms through which infection negatively affects outcomes in patients with intracerebral hemorrhage (ICH).

**Methods:**

A prospective observational study in patients with acute ICH classified by the occurrence of in-hospital infection within 7 days and outcomes at 6 months, good outcome defined as an improvement of > 10 points or > 50% in NIHSS score and a mRS score 0–2. Immune cell-derived EVs were obtained from blood samples at 7 days by immunoprecipitation with anti-CD3 (T cells), anti-CD20 (B cells) and anti-CD14 (monocytes) antibodies. The protein content of the EVs was analyzed by data independent acquisition mass spectrometry. Differential abundance between groups was defined as fold-change ≥ 2 or ≤ 0.5 and *p* ≤ 0.05.

**Results:**

The study enrolled 44 patients: 17 (39%) infected, 14 (82%) with poor outcomes, and 27 (61%) with no infection, 12 (44%) with poor outcomes. There were 190 proteins with differential abundance in the EVs of infected patients, 6 relevant proteins associated with poor outcome and infection: in T cell-derived EVs PSME1 (involved in apoptosis), H2B1C and MTREX (involved in transcription regulation, DNA replication and DNA repair) were more abundant; in B cell-derived EVs, COHA1 (organization of extracellular matrix) was less abundant; and in monocyte-derived EVs, PCSK9 (cholesterol metabolism) and CMC1 (energy-related metabolic pathways) were less abundant.

**Conclusions:**

A cluster of proteins in immune system-derived EVs are involved in key biological pathways potentially linked to infection-related poor outcomes in patients with ICH.

**Supplementary Information:**

The online version contains supplementary material available at 10.1186/s12974-025-03635-9.

## Background

Intracerebral hemorrhage (ICH) accounts for approximately 15% of all strokes, with an incidence of 23 cases per 100,000 inhabitants/year and an associated mortality rate of about 50% [1]. Among survivors, more than 60% suffer sequelae that cause some degree of dependency [2]. Medical complications are major contributors to morbidity and mortality in patients with stroke, infections being one of the most frequent. In stroke patients, infection leads to worse neurological recovery, with poorer medium and long-term outcomes, and increased mortality rates. All this leads to an increase in healthcare costs, longer hospital stays and higher readmission rates [3].

Despite the high incidence of infection in patients with ICH ranging from 26% to 58% [4], the relationship between the two entities is still unclear. In particular, the mechanisms by which infection influences the course and may hamper neurological recovery in ICH patients are not well known. The study of circulating extracellular vesicles (EVs) has proven useful in the investigation of biological mechanisms underlying various diseases and could also be of help in this regard. EVs are nanometer-sized membrane vesicles secreted by all cell types that contain different molecules for intercellular signaling [5]. These molecules may vary in response to or reflect physiological or pathological conditions and therefore may serve as biomarkers of the underlying biological processes, also in neurological diseases [6]. In ICH, it has been shown that the protein content of circulating EVs may reflect evolutionary changes in the pathophysiology of the disease [7] as well as mechanisms related to brain repair [8, 9].

EV content may vary as a consequence of stress, cell damage or infectious processes [10, 11]. Thus, the content of EVs derived from immune cells such as monocytes, B cells and T cells may reflect not only the response of innate and adaptive immunity to infection but also, in the context of ICH, could open an interesting avenue to understanding the mechanisms responsible for poor outcomes associated with infections and to explore potential therapeutic targets. The differential expression of proteins in circulating EVs from immune cells in patients with ICH according to the occurrence of infections and to outcomes were investigated in this work.

## Methods

### Data availability

The mass spectrometry proteomics data have been deposited with the ProteomeXchange Consortium via the PRIDE [12] partner repository with the dataset identifier PRIDE: PXD054777.

### Study design

The study was approved by the La Paz University Hospital Clinical Research Ethics Committee (PI-3093). All the patients or their relatives signed an informed consent prior to inclusion in the study.

This clinical, prospective, and observational pilot study was conducted in accordance with the Strengthening the Reporting of Observational Studies in Epidemiology (STROBE) statement [13], ensuring adherence to recommended methodological and reporting standards. Male and female patients over 18 years of age, diagnosed with spontaneous supratentorial ICH within 24 h from symptom onset, admitted to the La Paz University Hospital Stroke Unit, Madrid, Spain, and treated according to current clinical recommendations between February 2019 and June 2022 were included in the study.

The exclusion criteria were: active infection at ICH onset, defined as the presence of clinical symptoms consistent with infection at any site, accompanied by fever and objective evidence of systemic or localized inflammation, as determined by blood tests, urine analysis, radiological findings (e.g., chest X-ray), or positive testing for COVID-19 or influenza; traumatic ICH; infratentorial location; signs of herniation or hydrocephalus; mild (National Institute of Health Stroke Scale (NIHSS) score < 4) or very severe (NIHSS score > 25) neurological deficit; history of previous stroke; previous deficit preventing accurate assessment of outcomes (modified Rankin Scale (mRS) score > 1); severe concomitant disease with short-term life expectancy; participation in clinical trials; and refusal to participate.

Demographic and clinical data were registered as well as the volume and location of the hematoma on a cranial CT on admission and at 5 to 7 days of follow-up. Venous blood samples were collected at 7 days from stroke onset. Clinical deficit was quantified on admission and at follow-up using the NIHSS score, and functional status was scored using the mRS. Occurrence and timing of infection during hospital admission was also recorded. Patients completed follow-up visits at 7 days, 3 months and 6 months after stroke onset.

At the end of follow-up, patients were classified according to whether or not they presented infection within the first 7 days from stroke onset (with the same criteria as previously defined: confirmatory clinical signs, presence of fever, leukocytosis, elevated CRP, or microbiological isolation) and to evolution. Good outcome was defined as an improvement of > 10 points or > 50% with respect to baseline NIHSS and functional independence (ERm 0–2) at 6 months.

### Blood extraction and extracellular vesicles isolation

Blood samples were centrifuged at 3,000 g for 15 min at 4 °C and collected serum was stored at − 80 °C until analysis.

EVs were isolated from serum using the ExoQuick isolation kit (System Biosciences, USA) following the manufacturer’s instructions and re-suspended in 100 µL of phosphate buffered saline (PBS).

EVs from T and B cells and from monocytes were isolated by immunoprecipitation with biotinylated antibodies (Invitrogen, USA): anti-CD3 to obtain T cell-derived EVs; anti-CD20 to obtain B cell-derived EVs; and anti-CD14 to obtain monocyte-derived EVs. Samples were then incubated with Pierce Streptavidin Plus UltraLink Resin (Thermo Fisher Scientific, USA). The beads were sedimented by centrifugation at 4,500 g for 5 min to obtain the EVs of interest, resuspended in BSA and Tris-HCl [14], and stored at − 80 °C until analysis.

### Characterization of extracellular vesicles

EVs were characterized by three different methods according to the latest Minimal Information for Studies of Extracellular Vesicles (MISEV) guidelines, as described below [15].

### Western blot

Western blot was performed to identify the surface proteins of EVs in a 10% sodium dodecyl sulphate-polyacrylamide gel (SDS-PAGE) for electrophoresis. Anti-CD9 (1:250), anti-CD81 (1:250), anti-CD63 (1:500), and anti-Alix (1:500) (Invitrogen, USA) were used; and anti-albumin (1:1000) to determine the purity of the sample (Invitrogen, USA) followed by an HRP-secondary antibody (1:750) (Abcam, UK). Bands were visualized using ECL Pierce chemiluminescence following the manufacturer’s instructions (Thermo Fisher Scientific, USA) in an Uvitec-Cambridge image acquisition system (Uvitec, UK).

### Nanoparticle tracking analysis (NTA)

The NanoSight NS500 nanoparticle analyzer (Malvern Instruments, UK) was used to assess the size distribution and concentration of the purified EVs derived from the 3 cellular origins. The samples were diluted in PBS, and particle movement was captured in three 60-second videos at a detection threshold of 3. These recordings were then analyzed using the NTA Software 2.3 version (Malvern Instruments, UK).

### Transmission electron microscopy (TEM)

The size and shape of the isolated EVs was examined using transmission electron microscopy (TEM). The EVs were fixed (5 min) on a drop of PFA 2%, washed two times with Milli-Q, and negatively stained with 2% aqueous uranyl acetate (1 min). The EVs were observed through a Tecnai 12 FEI electron microscope operated at 120 kV, and the images were recorded with a CCD (charged coupled device) FEI Ceta camera.

### Proteomic analysis

A data-dependent acquisition (DDA) mass spectrometry qualitative analysis and protein quantification by sequential window acquisition of all theoretical fragment ion mass spectra (SWATH-MS) data independent acquisition (DIA) was performed on EVs at 7 days to determine the specific protein content in the study groups. Samples were divided into three pools per study group prior to SWATH-MS analysis, except for the infection and good outcome group, which consisted of three individual samples. Pools were constructed based on hematoma volume and NIHSS score ensuring that each pool included patients with comparable clinical severity and hematoma burden. Pooling is a widely accepted strategy in discovery proteomics, as it reduces inter-individual biological variability and enhances the detection of disease-associated molecular signatures, rather than differences driven by patient-specific heterogeneity [16, 17].

To perform the qualitative and quantitative protein identification, an equal amount of protein (100 µg) was processed. Peptides of 4 µg were digested and separated using reverse phase chromatography. The gradient was created using the micro-liquid chromatography system nano LC 400 (SCIEX, USA) coupled to a high-speed Triple TOF 6600 mass spectrometer (SCIEX, USA) with a microflow source using a DDA method. Proteins were identified using a human-specific Uniprot database [18]. For this analysis, only those proteins with an FDR < 1% (99% protein confidence) were selected. For the relative quantification by the SWATH-MS DIA method, a spectral library grouping each EV type in a pool was built. Next, 4 µg of each sample were run in the TripleTOF 6600 using a SWATH-MS acquisition method [19]. A detailed explanation of the proteomic acquisition is provided in the supplementary material.

Information on the function and biological pathways in which the proteins are involved was obtained by consulting the PANTHER (Protein Analysis THrough Evolutionary Relations) system [20] and the Reactome pathway database [21].

### Validation of selected proteins by western blot

After the proteomic study, the proteins found to be related to infection and outcomes were verified by triplicate using western blot with their specific antibodies (Thermo Fisher Scientific, USA): anti-PSME1 (1:1000); anti-SKIV2L2 (1:1000); anti-H2B (1:1000); anti-PCSK9 (1:500); anti-CMC1 (1:1000); and anti-COL17A1 (1:1000). The procedure for western blot analysis was the same as described for the characterization of the EVs.

### Statistical analysis

Clinical data are expressed as mean and standard deviation (SD), median and interquartile range (IQR), and percentage as appropriate. Normality was checked using the Shapiro-Wilk test. Normally distributed variables were compared using ANOVA for each factor and Tukey’s *post hoc* test. For variables with non-normal distribution, groups were compared using the Kruskal-Wallis test followed by the Mann-Whitney test. Fisher’s exact test was used for percentage comparisons. *P*-values ≤ 0.05 were considered significant. All analyses were performed using the statistical software SPSS 27 (IBM, USA) and the figures using GraphPad Prism 8 (GraphPad Software, USA).

The results from the proteomics studies were analyzed using SWATH-MS principal component analysis (PCA) and cluster analysis, R 3.5.3 (R Core Team, Austria) and “base,” “stats,” “gplots,” “Hmisc,” “dplyr,” and “car” packages. Student’s t test for comparing means between samples was applied. Proteins showing differences between study groups larger than 2-fold change (FC) (greater abundance) or below 0.5 FC (lower abundance) at the significance level of *p* value < 0.05 were considered as having a differential expression. PCA analyses were performed using R 4.4.3 (R Core Team, Austria) and RStudio 2024.12.1 (Posit PBC, USA). PCA was used to visualize variability in proteomic data across the comparisons. To minimize technical variability and ensure that the observed separation reflects genuine biological differences, batch effect correction was applied from the “limma” package [22], correcting for pool, cell type, and outcome. PCA was performed using “prcomp*”* with standardization to balance the contribution of all proteins. Explained variance was calculated, and principal component (PC) 1 vs. PC2 was plotted using the “ggplot2” package (Posit PBC, USA).

To explore the biological pathways associated with differentially expressed proteins, functional enrichment analyses were performed using R v. 4.5.1 (R Core Team, Austria), RStudio 2024.12.1 (Posit PBC, USA), and Bioconductor packages. The analysis was conducted separately for proteins with higher and lower abundance detected in the three cell populations. Enrichment analyses were performed with the “clusterProfiler” and “ReactomePA” packages. Gene Ontology (GO) domains (Biological Process and Cellular Component) were assessed using the enrichGO() function, and Reactome pathway analysis was conducted using enrichPathway(). The Benjamini–Hochberg method was applied to adjust p-values, and terms with an adjusted FDR < 0.05 were considered statistically significant. Visualizations were generated with ggplot2, displaying − log₁₀(FDR) values and gene counts for each enriched term.

Post-hoc effect size estimation was performed in R v. 4.5.1 (R Core Team, Austria) using a “pwr” package. For each protein, group Log2 means and standard deviations (SD) obtained from three pooled biological replicates per condition and Cohen’s d was computed as the standardized mean difference between groups using the pooled SD weighted by sample size. Given the limited number of replicates, Hedges’ g was used as a small-sample bias-corrected estimate of effect size. When SD in one group was zero, the corresponding effect size was considered not estimable.

## Results

**Fig. 1:**
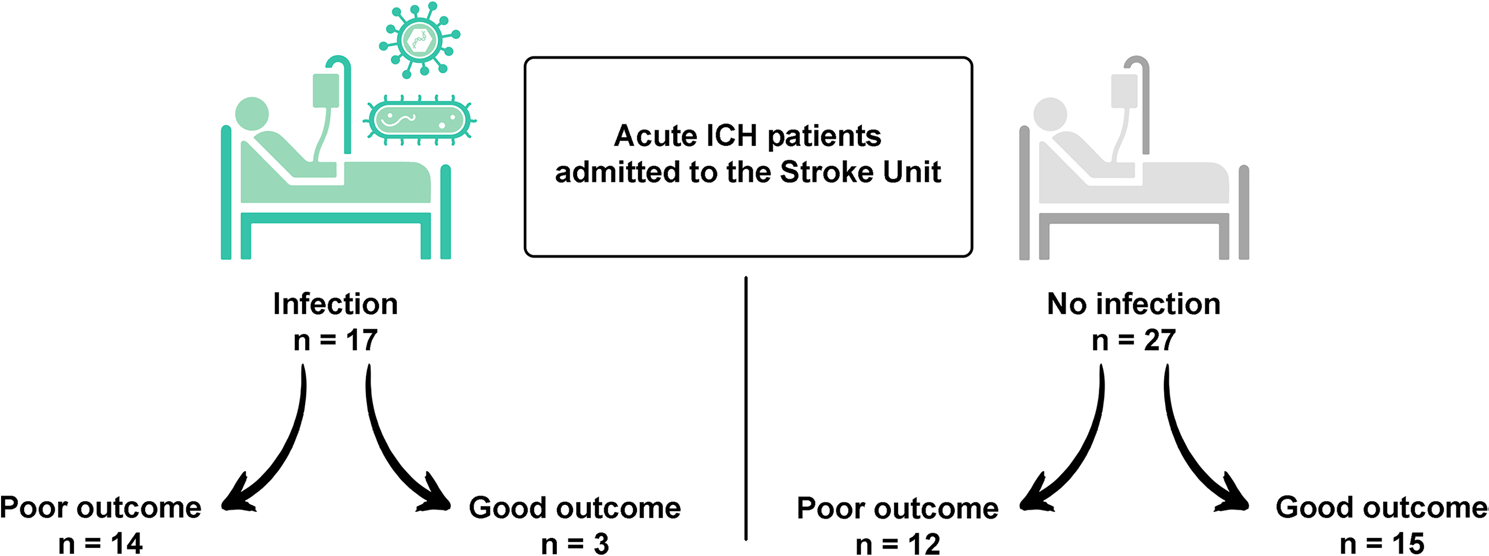
Patient’s classification in the study

The study included 44 patients with ICH of which 17 (39%) developed an infection during hospital admission (Fig. 1). Most of the infections were respiratory (in 10 patients), there were 4 urinary tract infections, 2 patients both (urinary tract infection and respiratory infection) and in one patient staphylococcus epidermidis was isolated in cerebrospinal fluid from external ventricular drainage.

There were no differences in demographics and risk factors between patients who suffered an infection and those who did not, except for age, those with infection being older (73 vs. 64 years, *p* = 0.045). Nor were there differences in clinical severity, hemorrhage volume, vital signs or laboratory data on admission. However, patients who developed infection had a worse neurological course, as shown in Table 1. Table 2 shows the differences between patients with good and poor outcomes in the infection and no-infection groups. Although there were no differences in clinical severity on admission nor in the baseline ICH volume between infected and non-infected patients, patients with poor outcomes had larger hematoma volumes and higher NIHSS scores on admission in both groups. However, among patients with poor outcomes, there were no differences in hemorrhage volume or severity between those who developed an infection and those who did not (Table S1).

**Table 1:** Demographic data, risk factors, and clinical data

	Infection	No infection	
	*n* = 17	*n* = 27	*p* value
Age, years, mean (SD)	73 (10.8)	64 (13.1)	**0.045**
Sex, male, *n* (%)	18 (66.8)	18 (66.7)	0.526
Hypertension, *n* (%)	21 (77.8)	21 (77.8)	0.455
Diabetes mellitus, *n* (%)	6 (22.2)	6 (22.2)	0.455
Dyslipidaemia, *n* (%)	11 (40.7)	11 (40.7)	0.354
Atrial fibrillation, *n* (%)	2 (11.8)	4 (14.8)	0.730
Baseline systolic blood pressure, mean (SD)	177.4 (24.9)	180.1 (37.2)	0.749
Baseline diastolic blood pressure, mean (SD)	94.6 (11.2)	101.8 (23)	0.249
Baseline glycemia, mean (SD)	126.2 (23.9)	128.42 (35)	0.655
Baseline O_2_ saturation, mean (SD)	94.9 (2.7)	96.2 (2.7)	0.072
Baseline NIHSS, median (IQR)	12.0 (11.3)	10.5 (8.3)	0.256
Baseline ICH volume mm^3^, mean (SD)	15.9 (10.7)	10.9 (11.1)	0.101
7 d NIHSS, median (IQR)	10.5 (14.5)	4.5 (8.5)	**0.004**
7 d ICH volume mm^3^, mean (SD)	17.0 (11.9)	10.0 (12.1)	0.056
3mo NIHSS, median (IQR)	3.5 (9.0)	1 (4.5)	**0.047**
3mo mRS, median (IQR)	3 (0)	2 (2.8)	0.067
6mo NIHSS, median (IQR)	3 (5)	1 (4.3)	0.086
6mo mRS, median (IQR)	3 (0)	2 (2)	0.075

Bold *p* numbers indicate *p* < 0.05

*Abbreviations:IQR* interquartile range, *mRS* modified Rankin Scale, *NIHSS* National Institutes of Health Stroke Scale score, *SD* standard deviation

**Table 2:** Demographic data, risk factors, and clinical in infected and non-infected patients according to outcomes

	Infection		*p *value	No infection		*p* value
	Poor outcome (*n* = 14)	Good outcome (*n* = 3)		Poor outcome (*n* = 12)	Good outcome (*n* = 15)	
Age, years, mean (SD)	72 (11.2)	74 (10.8)	0.752	72 (9)	57 (11.9)	**0.005**
Sex, male, *n* (%)	9 (64.3)	0 (0)	0.082	7 (58.3)	11 (73.3)	0.488
Hypertension, *n* (%)	12 (85.7)	3 (100)	1	10 (83.3)	11 (73.3)	0.622
Diabetes mellitus, *n* (%)	2 (14.3)	0 (0)	1	3 (25.0)	12 (80)	1
Dyslipidaemia, *n* (%)	10 (71.4)	0 (0)	0.051	6 (50.0)	5 (33.3)	0.452
Atrial fibrillation, *n* (%)	2 (14.3)	0 (0)	1	4 (33.3)	0 (0)	**0.028**
Baseline systolic blood pressure, mean (SD)	174.5 (23.2)	190.7 (33)	0.257	171.9 (36.6)	185.5 (37.9)	0.279
Baseline diastolic blood pressure, mean (SD)	93.1 (9.9)	101.3 (16.7)	0.528	98.9 (30.9)	103.7 (16.7)	0.359
Baseline glycemia, mean (SD)	127.9 (25.4)	118 (16.5)	0.801	141.3 (45.3)	117.4 (18.1)	0.129
Baseline O_2_ saturation, mean (SD)	95.5 (2.1)	91 (1.4)	0.55	95.8 (2.8)	96.6 (2.4)	0.588
Baseline NIHSS, median (IQR)	17.5 (11.3)	13 (---)	0.344	19.5 (11.5)	8 (6)	**0.001**
Baseline ICH volume mm^3^, mean (SD)	18.1 (10.5)	5.5 (3.2)	**0.032**	17.3 (14.2)	5.8 (3.1)	0.067
7 d NIHSS, median (IQR)	18 (12)	5 (---)	**0.031**	11(8)	4 (3)	**0.0001**
7 d ICH volume mm^3^, mean (SD)	19.2 (11.8)	6.8 (5.9)	0.101	17.3 (16.2)	4.7 (1.8)	**0.018**
3mo NIHSS, median (IQR)	4 (17)	2.5 (---)	0.319	9 (10.5)	1 (2)	**0.0001**
3mo mRS, median (IQR)	3 (1)	1.5 (---)	**0.016**	4 (1.3)	2 (1)	**0.0001**
6mo NIHSS, median (IQR)	6 (11)	1 (---)	0.077	7 (6.5)	0 (1)	**0.0001**
6mo mRS, median (IQR)	3 (1.5)	1 (---)	**0.005**	4 (1)	1.5 (1.3)	**0.0001**

Bold *p* numbers indicate *p* < 0.05

*Abbreviations*: *IQR* interquartile range, *mRS* modified Rankin Scale, *NIHSS* National Institutes of Health Stroke Scale score, *SD* standard deviation

### Characterization of extracellular vesicles

Western blot analysis confirmed by triplicate the presence of EV-specific markers (CD9, CD81, CD63 and Alix) in all samples (Figure S1A). The morphology and size distribution of EVs were confirmed by TEM and NTA (Figure S1B y S1C).

### Proteomic analysis of extracellular vesicles content

**Fig. 2:**
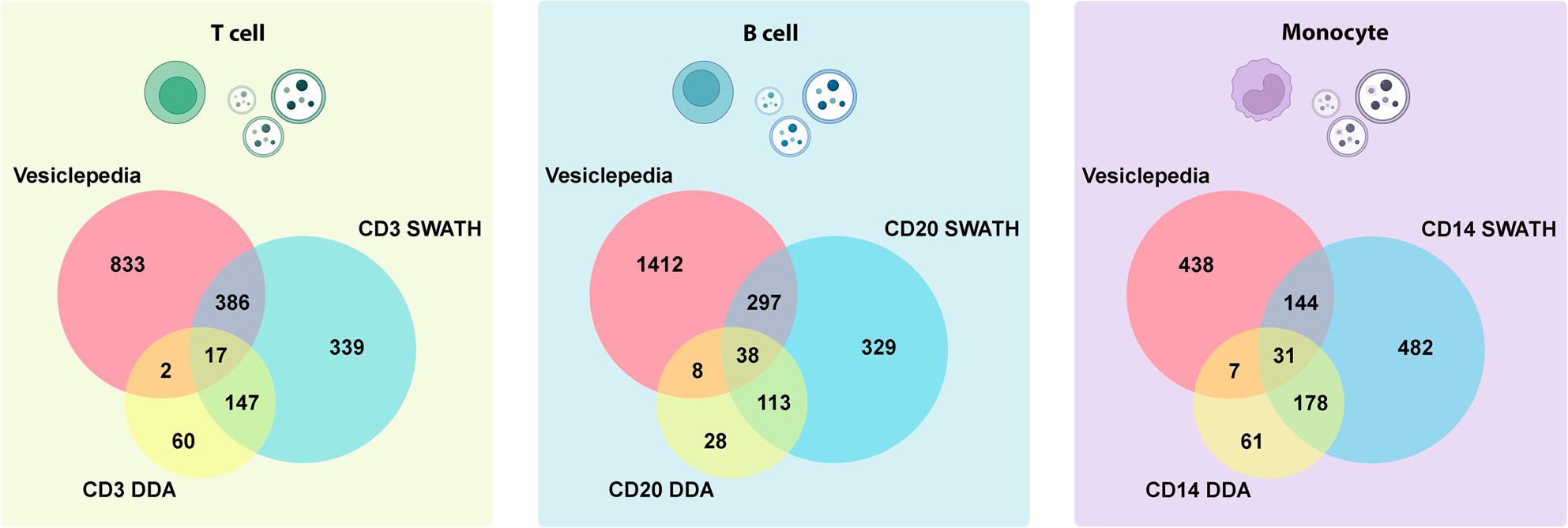
Proteins found in DDA and SWATH analysis. DDA (yellow) and SWATH (blue) proteins of each immune cell-derived EVs were compared to the proteins described previously in the same immune cells in vesiclepedia (red)

To confirm the origin of immunoisolated EVs, SWATH and DDA results of each EVs subpopulation were compared to a database of proteins located in T cells, B cells and monocyte-derived EVs (vesiclepedia) [23], as shown in Fig. 2.

**Fig. 3:**
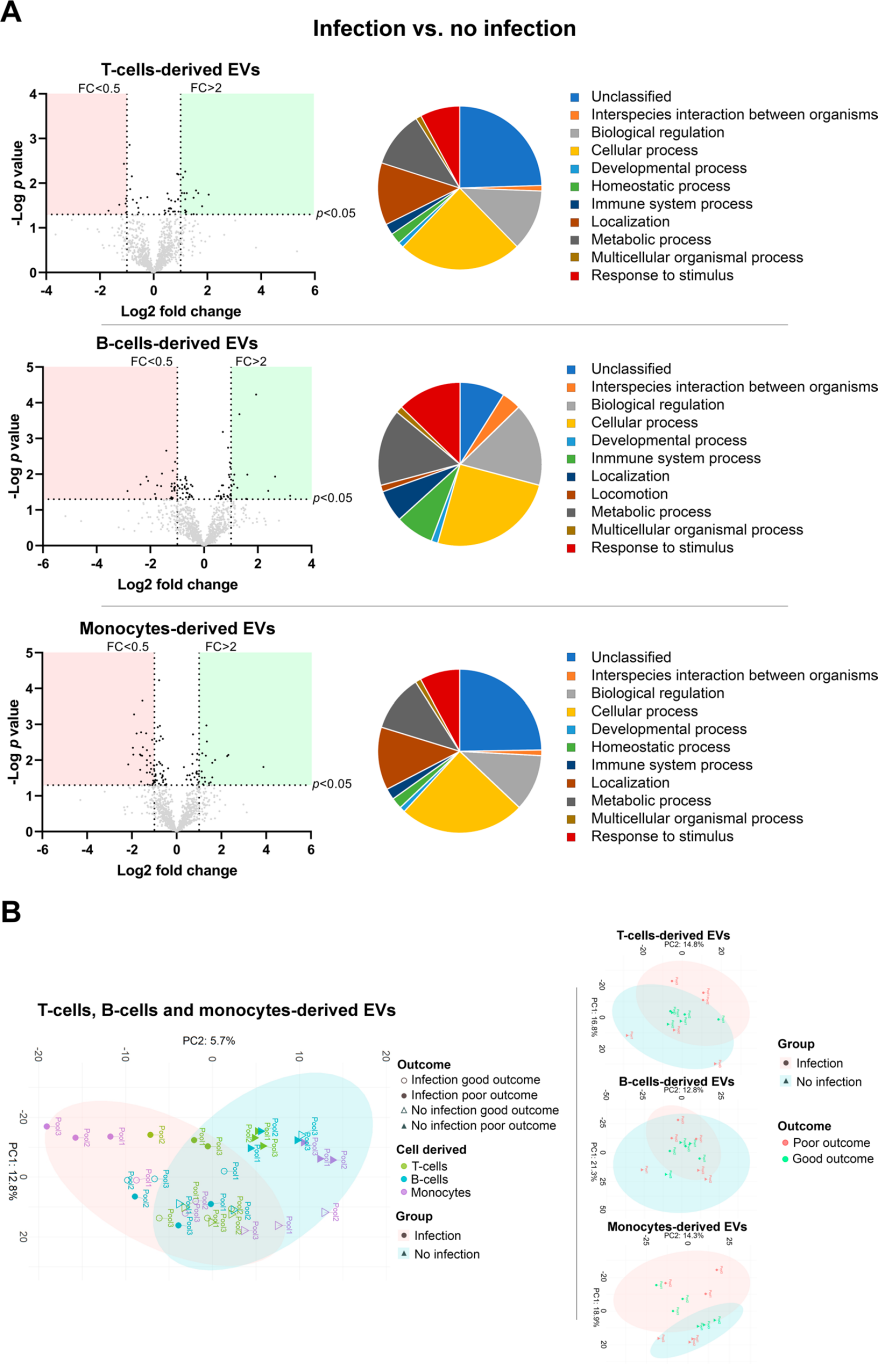
Protein abundance and biological processes in T cell-, B cell- and monocyte-derived EVs in infected vs. non-infected patients. **A** The volcano plots show a comparison of the various proteins present in EVs in each study group. Proteins with higher abundance are shown in the green areas and those with lower abundance in the red areas. The pie chart shows the relative representation of the most relevant biological processes in which the differential proteins are involved. **B** PCA graphs of the proteomic samples analyzed

To identify the proteins associated with infection-related poor outcomes, differences in the proteome of EVs from patients who developed infections with those who did not were analyzed in a first step. Proteomic analysis revealed 109 proteins with differential abundance in the various immune cell-derived EVs. PCA analysis demonstrated a good separation of their PC between infection and no infection, particularly in T cells and monocyte-derived EVs. These findings, together with the biological processes and pathways in which these proteins are involved, are summarized in Fig. 3 and Table S2.

**Fig. 4:**
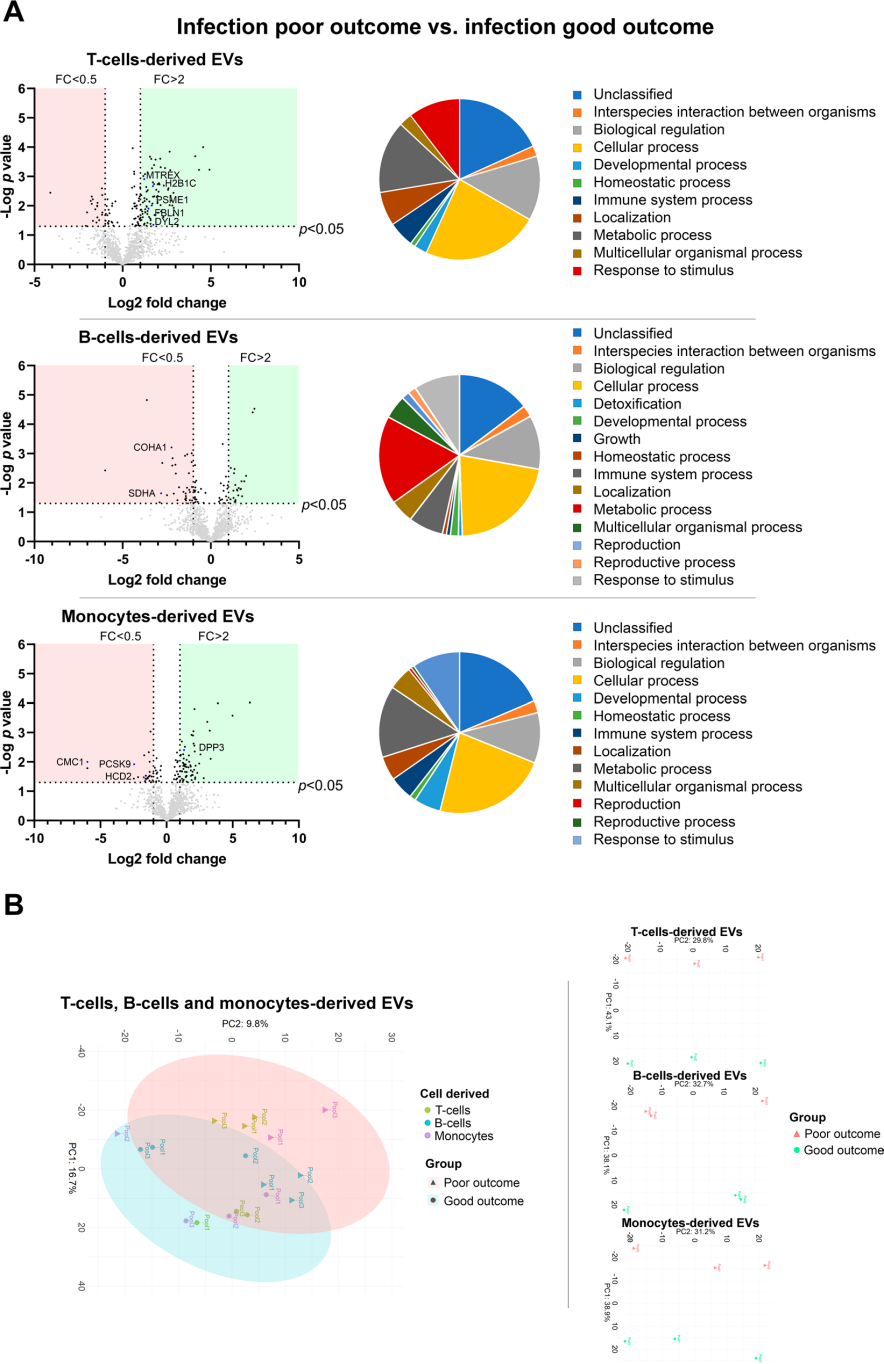
Protein abundance in T cell-, B cell- and monocyte-derived EVs and their biological processes in infected patients depending on outcomes. **A** The volcano plots compare the various proteins by group. Proteins with higher abundance are shown in the green areas and those with lower abundance in the red areas. The pie chart shows the relative representation of the most relevant biological processes in which the differential proteins are involved. **B** PCA graphs of the proteomic samples analyzed

To investigate whether any of these differential proteins may be related to the mechanisms by which infection is associated with poor clinical outcomes, protein abundance in the EVs from infected patients were compared with those with good and poor outcomes. Of the 109 proteins found to be associated with infection, 5 in T cell-derived EVs (PSME1, MTREX, H2B1C, FBLN1 and DYL2), 2 in B cell-derived EVs (SDHA and COHA1) and 4 in monocyte-derived EVs (DPP3, PCSK9, HCD2 and CMC1) showed differential abundance according to outcome. In the PCA analyses, the overall PCA plot showed a good separation of the principal components, with only one poor outcome monocyte sample falling within the good outcome ellipse. Additionally, each cell type demonstrated excellent separation between outcome samples (Fig. 4).

**Fig. 5:**
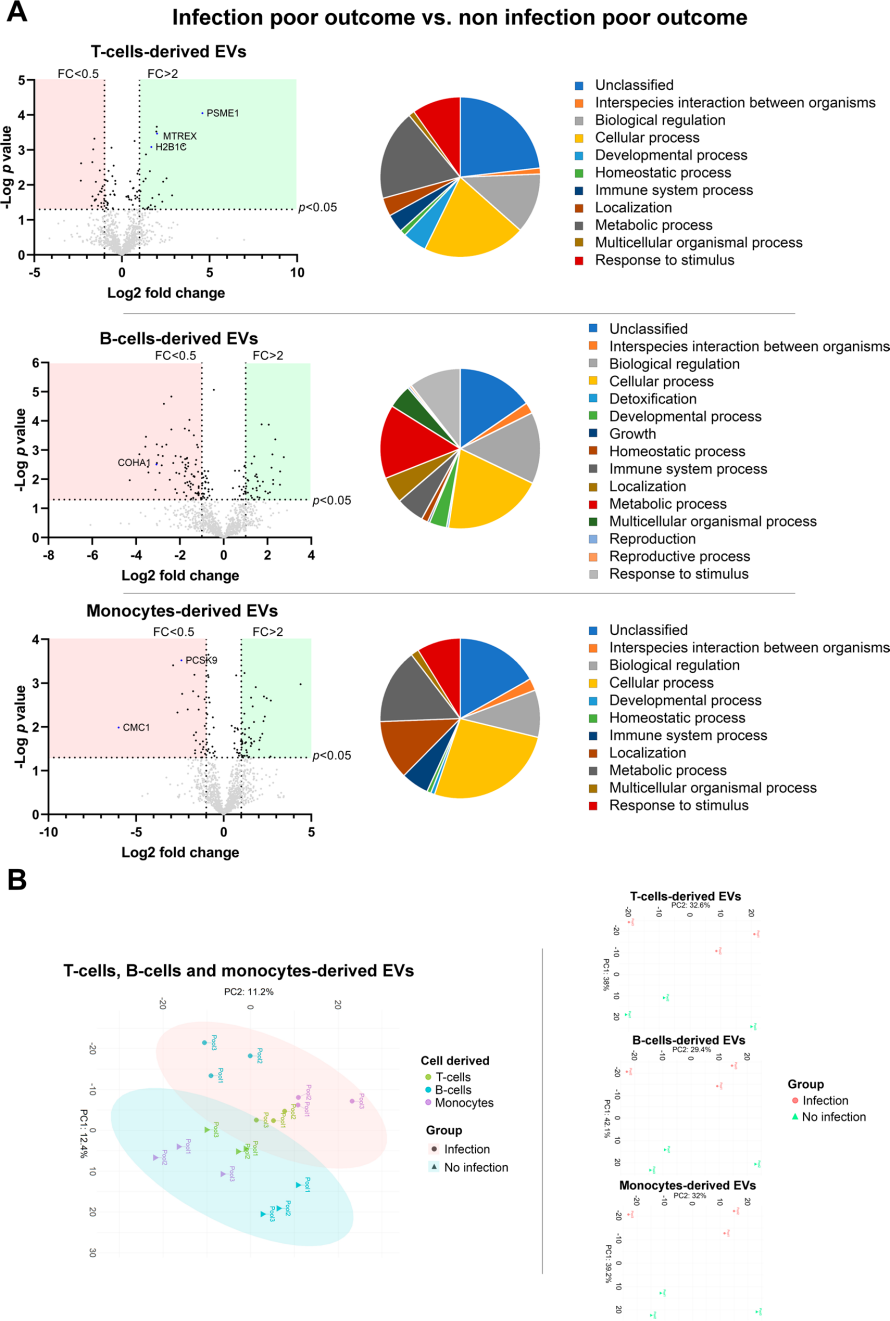
Protein abundance in T cell-, B-cells and monocytes-derived EVs and their biological processes in patients with poor outcomes depending on the development of infection. **A** The volcano plots compare the various proteins by group. Proteins with higher abundance are shown in the green areas and those with lower abundance in the red areas. The pie chart shows the relative representation of the most relevant biological processes in which the differential proteins are involved. **B** PCA graphs of the proteomic samples analyzed

Finally, to determine whether the difference in protein abundance associated with poor outcomes in infected patients was in fact related to the infection and not to the hemorrhage itself, the formerly described 11 proteins in the various EVs were compared between patients with poor outcomes with infection and those with poor outcomes without infection. Among these proteins, 6 showed differential abundance. In patients with poor outcome and infection, PSME1, MTREX, and H2B1C were more abundant in T cell-derived EVs; COHA1 was less abundant in B cell-derived EVs; and PCSK9 and CMC1 were less abundant in the monocyte-derived EVs. Again, PCA analysis demonstrated an excellent separation of each principal component between Infection and no infection, as well as between individual cell type isolations (Fig. 5). The proteins with differential abundance were validated by Western blot by triplicate (Figure S2).

**Fig. 6:**
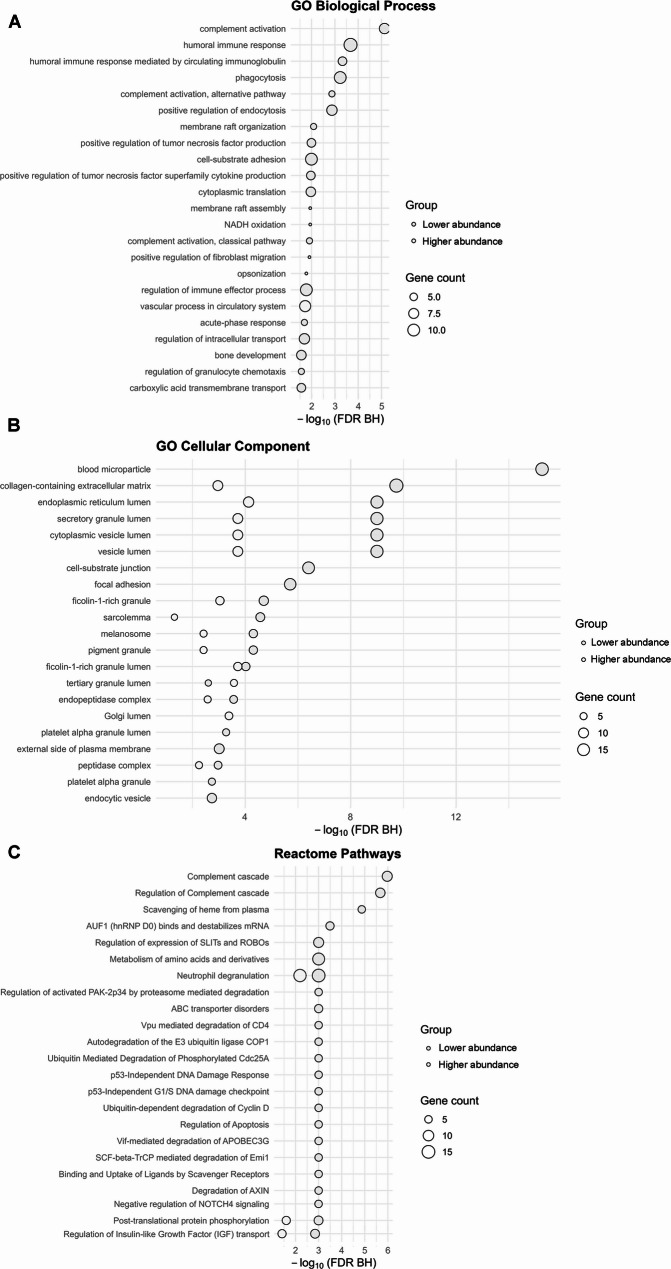
Enrichment analysis on the dysregulated proteins. **A** Biological processes. **B** Cellular component. **C** Reactome pathways. Lower abundance proteins (red) show significant immune-related enrichment, while higher abundance proteins (green) display limited functional clustering. Circle size: gene count; FDR: false discovery rate (Benjamini-Hochberg correction)

Biological processes analysis revealed striking differences between dysregulated proteins. Lower abundance proteins in infected patients showed significant enrichment in multiple immune-related pathways, particularly complement activation, humoral immune response, and regulation of endocytosis. In contrast, higher abundance proteins showed minimal enrichment, with only vascular processes in the circulatory system being significantly represented. Cellular component analysis demonstrated that lower abundance proteins were predominantly associated with extracellular compartments (blood microparticles, extracellular matrix, vesicle lumen), while higher abundance proteins were enriched in intracellular granules (specific and tertiary granules) and endoplasmic reticulum. Reactome pathway analysis confirmed the immune dysfunction pattern, with lower abundance proteins enriched in complement cascade and heme scavenging pathways, contrasting with higher abundance proteins involved in protein degradation and cellular signaling (Fig. 6).

The post-hoc power analysis showed the robustness of the proteomic findings. The key differentially abundant proteins demonstrated large effect sizes (*d*/g = 4–7; Table S3), indicating that, despite the limited sample size, the probability of detecting true differences was high. Post-hoc power could not be calculated for the CMC1 protein because spectral values were zero in one of the comparison groups, precluding effect size estimation.

## Discussion

Our pilot study provides novel evidence that patients with ICH who develop infections exhibit a distinct protein profile in circulating immune cell-derived EVs and that some of these proteins appear to be specifically associated with poorer outcomes in infected patients. The biological pathways in which these proteins are involved may be related to such outcomes.

The immune system is increasingly regarded as a pivotal factor in the pathophysiology of ICH, not only in the various steps of brain damage, but also in processes related to brain repair [24, 25]. Moreover, the immune system is a key factor in the development of infections, one of the most frequent complications after ICH that worsens patients’ clinical condition. Several studies have described changes in the immune system after a stroke in terms of lymphopenia, reduced monocyte count, T and NK cell dysfunction and changes in serum levels of certain cytokines, favoring post-stroke infections and hampering recovery [25, 26]. However, little is known about how immune dysregulation might be involved in the cross-talk between infections and the mechanisms underlying brain damage and repair after ICH. To elucidate these mechanisms, the protein content of circulating EVs from immune system cells after ICH and infection were studied, rather than the number of immune cells or cytokine levels, considering EVs as essential for intercellular communication, and that their content reflects the functional status of their cells of origin. Our findings may help in understanding the processes triggered by infection that worsen the clinical outcome of these patients, as a first step towards further research into targeted therapies.

The time point of 7 days was selected for analysis, since most infections occur within the first week following stroke onset [3]. Additionally, the first week post-stroke is characterized by a systemic response aimed at tissue repair [24]. Therefore, examining the relationship between infection and recovery with biomarkers expressed at 7 days post-stroke may provide insights into the cross-talk between these mechanisms during a critical period of immune activation and repair processes.

In our first approach, 109 proteins were found with differential abundance between patients who developed infection and those who did not. These proteins are involved in numerous important biological functions such as regulation of cellular processes, localization, metabolism, biological regulation and response to stimulus that are probably triggered by the infection and that reflect the systemic response to it.

To examine the potential involvement of these proteins in the mechanisms linking infection to poor outcomes, an analysis comparing the proteome in EVs from patients who developed infections and had poor outcomes to those who developed infections but had better functional recovery was conducted. This analysis identified 11 differential proteins associated with poorer outcomes. This analysis suggests that proteins that are most probably associated with the poor outcome related to the infection and not to the ICH itself were identified. With the intention to confirm this data the former 11 proteins in EVs from patients with poor outcomes and infection were compared to patients with poor outcomes and no infection. Six of these proteins were differentially associated with infection and poor outcomes and were therefore proposed as related to the mechanisms by which infection alters neurological recovery after ICH.

Among these proteins, three were more abundant in T cell-derived EVs: PSME1, MTREX and H2BC1. Recent studies show that T lymphocytes induce apoptosis post-ICH injury while T lymphocyte depletion in animal models has been shown to decrease perihematomal edema and improve functional outcomes [27]. T cell-derived EVs have also been described as regulating the functions of other immune cells as part of an immune response and inducing apoptosis [28]. Activation of T lymphocytes as a consequence of infection could modify the proteome of their EVs towards a pro-apoptotic state and therefore increase the deleterious role of these lymphocytes in the injury cascade after ICH, thus hampering recovery. Of the proteins found, PSME1 (proteasome activator complex subunit 1) is essential to immunoproteasome assembly for efficient antigen processing and participates in mechanisms of programmed cell death, among other functions [21, 29]. Our results suggest PSME1 is one of the mediators of the proapoptotic signature of T cell-derived EVs after infection. H2BC1 (histone H2B type 1-C) had never been identified before in EVs. This is a core component of the nucleosome participating in transcription regulation, DNA repair, DNA replication and chromosomal stability [18, 21]. Finally, MTREX (exosome RNA helicase MTR4) participates in RNA metabolism and in the response to DNA damage [18, 21]. The fact that these proteins are over-abundant in infected patients with poor outcomes may reflect the need to enhance these repair mechanisms to mitigate the greater damage triggered by infection. Considering the functions of all these proteins as a whole, our data suggests that, in patients with cerebral hemorrhage who develop an infection, there is an activation of the immune system with proteins carried in T cell EVs that try to repair the damage or induce apoptosis if the damage persists.

Macrophage-derived EVs have been implicated in the modulation of inflammatory processes and recent studies have shown that macrophage-derived EVs contribute to the progression of atherosclerosis, diabetes and heart disease [30]. In our work, less abundance of PCSK9 and CMC1 in monocyte/macrophage-derived EVs was found. This is the first time that these proteins have been identified in monocyte/macrophage-derived EVs.

Both proteins are involved in metabolic pathways [21]. PCSK9 (proprotein convertase subtilisin/kexin type 9) is a crucial protein in low-density lipoprotein (LDL) cholesterol (LDL-C) metabolism. PCSK9 induces lysosomal degradation of the LDL receptor in the liver, which is responsible for removing LDL-C from circulation [18]. Lower abundance of PCSK9 is associated with decreased levels of circulating LDL. Numerous studies have associated low LDL levels with increased risk of bleeding [31, 32]. Previous data from our group showed increased abundance of PCSK 9 in the circulating EVs of rats experiencing good outcomes in a preclinical study of ICH [8], which is in line with the present data showing lower abundance of PCSK9, particularly in macrophage-derived EVs in patients with poor outcomes and infection. All these data suggest that PCSK9 could be involved in recovery after ICH, maybe by regulating cholesterol metabolism as a key component of cellular architecture, although further studies are needed to unveil its role.

CMC1 (COX assembly mitochondrial protein homolog) is a conserved protein that localizes to mitochondria in human cells [18]. It is involved in the aspartate and asparagine metabolic pathway mediating the synthesis of aspartate and asparagine from glutamate and allowing the use of carbon atoms from these amino acids for glucose synthesis under fasting conditions [21]. Previous studies have linked macrophage-derived EVs to glucose metabolism and diabetes through their content in micro-RNAs [30]. Although further studies are needed to understand the role of CMC1 in macrophage-derived EVs, finding this protein at lower levels in patients with ICH and infection-related poorer outcomes suggests an uncoupling between the metabolic demands in stressful conditions and the bioavailability of glucose as an energy source, as related to poorer prognosis of these patients. This finding opens up an interesting avenue for further research.

Finally, lower levels of COHA1 (collagen alpha-1(XVII) chain) protein in B cell-derived EVs were found. Recent articles show that B cells-derived EVs are involved in antigen presentation, stimulate T cells and amplify the immune response [28]. Although there is a paucity of data on the participation of the B cell immune response in stroke, a role in favoring repair has been described in ischemic stroke [33]. COHA1 participates in the immune cascade by modifying the response of B, T and NK cells to pathogens and in immunoregulatory interactions between lymphoid cells and non-lymphoid cells but also participates in the organization of the extracellular matrix [18, 21]. This is essential after brain damage to preserve the neurovascular unit and to promote tissue repair [34]. To our knowledge, this is the first time this protein has been described in EVs. The lower abundance of COHA1 in B cell-derived EVs in ICH patients with infection and poor outcomes suggests that poorer reorganization of the extracellular matrix jeopardizes tissue repair, which is in line with the worse outcomes in these patients. More studies are needed to confirm our results and to investigate the role of COHA1 in recovery.

Enrichment analysis suggests a mechanistic link between infection and hemorrhagic complications through immune pathway dysregulation. The significant lower abundance of complement cascade and humoral immune components indicated that infection induces immunodeficiency in patients with poor outcome. Given the complement system’s role in maintaining endothelial integrity and regulating coagulation [35], its suppression may directly contribute to vascular fragility and hemorrhage risk. The predominant extracellular localization of lower abundance proteins supports impaired blood-brain barrier function and disrupted neurovascular unit integrity. Meanwhile, the limited higher abundance of proteins associated with vascular processes and granulocyte granules suggests an activated but misdirected inflammatory response. This dysregulated immune landscape may explain why certain infected patients progress to worse hemorrhagic events despite standard therapeutic interventions. Future mechanistic investigations, including targeted functional assays, are needed to confirm these pathways’ causal roles in determining how immune dysregulation influences ICH outcomes in infected patients and to identify potential therapeutic targets.

## Limitations

The main limitation of our study is the small sample size which hinders stratification of patients by prognostic factors or specific infection phenotypes for a more detailed and robust analysis of changes in the proteomic signature. In particular, there were few patients in the infection and good outcome group. While 60% of the patients of the global cohort had a poor outcome, the presence of infection significantly worsened the prognosis, with more than 80% experiencing a poor outcome. Despite similar ICH volumes and comparable clinical severity upon admission between patients with and without infections, patients with poorer outcomes had greater clinical severity and hematoma volume in both groups. Bearing in mind that initial severity and size of hematoma are the main prognostic factors in ICH, an effect on the results cannot rule out. To mitigate this, a differential analysis of the proteins was performed, among those with poor outcomes with and without infection, that showed no differences in hematoma volume or NIHSS score, although larger samples would be necessary. Additionally, it would be of interest to analyze the protein content at different time points to identify earlier biomarkers that could help predict which patients are at risk of developing an infection and to provide further data on possible mechanisms involved in brain damage and repair. Finally, the present work does not include direct functional assays of the proteins of interest which limits our ability to establish causal mechanistic links with the observed outcomes. Therefore, further studies will be required to elucidate their specific biological roles. Despite these limitations, our exploratory study identified immune-derived proteins in extracellular vesicles that may be related to worse clinical course of patients with intracerebral hemorrhage who develop infection, generating new hypotheses and paving the way towards the search for therapeutic targets to improve the outcome of these patients.

## Conclusion

This study presents, for the first time, a cluster of proteins identified in immune cell-derived EVs associated with infection and poor prognosis in patients with ICH. These proteins are involved in key biological pathways, and alterations in their regulation appear to be related to the poorer outcomes observed in patients who develop infections.

## Supplementary Information

Supplementary Material 1. [36].

Supplementary Material 2: Table S1. Demographic data, risk factors, and clinical in patients with poor outcome with and without infection. Table S2. Proteins in T-cells, B-cells and monocytes-derived EVs in infected vs. non infected patients. Table S3. Statistical power (Cohen’s d and Hedges’ g) of selected proteins. Figure S1. Characterization of T-cells, B-cells and monocytes-derived EVs. Figure S2. Western blot for validation of the proteins found with differential abundance.

Supplementary Material 3.

Supplementary Material 4.

## Data Availability

The mass spectrometry proteomics data have been deposited with the ProteomeXchange Consortium via the PRIDE [12] partner repository with the dataset identifier PRIDE: PXD054777.

## Abbreviations

ICH: Intracerebral hemorrhage
EVs: Extracellular vesicles
STROBE: Strengthening the Reporting of Observational Studies in Epidemiology
NIHSS: National Institute of Health Stroke Scale
mRS: Modified Rankin Scale
MISEV: Minimal Information for Studies of Extracellular Vesicles
SDS-PAGE: Sodium dodecyl sulphate-polyacrylamide gel
DDA: Data-dependent acquisition
SWATH-MS: Sequential window acquisition of all theoretical fragment ion mass spectra
DIA: Data independent acquisition
PANTHER: Protein Analysis THrough Evolutionary Relations
SD: Standard deviation
IQR: Interquartile range
PCA: Principal component analysis
PC: Principal component
FC: Fold change
LDL: Low-density lipoprotein
LDL-C: LDL cholesterol
