# Supplementary material for: Infection-driven proteomic signatures in immune cell–derived extracellular vesicles reflect hemorrhagic stroke outcome
Source: J Neuroinflammation. 2025 Dec 4;23:22. doi: 10.1186/s12974-025-03635-9 (PMC12822078; doi:10.1186/s12974-025-03635-9)
Supplement: Supplementary file 1 — Supplementary Material 1. [36]. [file 12974_2025_3635_MOESM1_ESM.docx]

**Supplementary methods S1. Detailed methodology of proteomic analysis**

- 2. **Immune Cell-derived extracellular vesicle samples processed for protein identification by LC-MS/MS using a DDA method and quantification by LC-MS/MS using a DIA-SWATH-MS method**

The EV proteins were loaded on a 10% SDS-PAGE gel. The run was stopped as soon as the front had penetrated 3 mm into the resolving gel [7,8]. The protein band was detected by staining with Sypro-Ruby fluorescent (Lonza, Switzerland), excised, and processed for in-gel, manual tryptic digestion, as described elsewhere [7,8]. Peptides were extracted with three 20-min incubations in 40 μL of 60% acetonitrile dissolved in 0.5% HCOOH. The extracted peptides were pooled, concentrated in a SpeedVac, and stored at −20 °C.

**LC-MS-MS analysis**

We separated 4 μl (aprox 4 μg) of digested peptides of all individual samples, employing reversed-phase chromatography. A gradient was created using a micro liquid chromatography system (Eksigent Technologies nanoLC 400, SCIEX) coupled to a high-speed Triple TOF 6600 mass spectrometer (SCIEX) using a micro flow source. The analytical column was a silica-based reversed-phase Chrom XP C18 150 × 0.30 mm, 3-mm particle size and 120 Å pore size (Eksigent, SCIEX) and the trap column a YMC-Triart C18 (YMC Technologies, Teknokroma) with a 3-mm particle size and a 120 Å pore size. This precolumn was switched on-line with the analytical column. The loading pump delivered solution A (0.1% formic acid in water) at 10 µL/min. The micro-pump generated a flow-rate of 5 µL/min under gradient elution conditions, using solution A (0.1% formic acid in water) and B (0.1% formic acid in acetonitrile). Peptides were separated by a 90-minute gradient ranging from 2% to 90% mobile phase B in DDA mode and 40-minute gradient in SWATH-MS (DIA) mode [Laso-García et al 2023 a and b]. Data acquisition was performed with a TripleTOF 6600 System (SCIEX, Foster City, CA, USA), using a data-dependent acquisition (DDA) method. Source and interface conditions were as follows: ion spray voltage floating (ISVF) 5500 V, curtain gas (CUR) 25, collision energy (CE) 10, and ion source gas 1 (GS1) 25. The instrument was operated with Analyst TF 1.7.1 software (SCIEX, USA). Switching criteria was set to ions greater than mass to charge ratio (m/z) 350 and smaller than m/z 1400 with a charge state of 2-5, mass tolerance 250 ppm, and an abundance threshold of more than 200 counts (cps). Former target ions were excluded for 15 s. The instrument was automatically calibrated every 4 hours using tryptic peptides from PepCalMix as the external calibrant.

**Qualitative data processing for DDA analysis**

After MS/MS analysis, data files were processed with ProteinPilot^TM^ 5.0.1 software from SCIEX, which uses the algorithm Paragon^TM^ for database search and Progroup^TM^ for data grouping. Data were searched using a human-specific Uniprot database (UniProt release 2022_05 Published on December 14, 2020; 20594 human proteins). The false discovery rate was determined using a nonlinear fitting method displaying only those results that reported a 1% global false discovery rate or better [7,8].

**Protein quantification by SWATH-MS (sequential window acquisition of all theoretical mass spectra)- DIA analysis**

**Creation of the spectral library**

To construct the MS/MS spectral libraries, and to get a good representation of the peptides and proteins present in all samples, pooled samples from each group were prepared using equal mixtures of the original samples. Next, 4 μL (4 μg) of each pool were separated into a micro-LC system Ekspert nLC425 (Eksigen, Dublin, CA, USA) using a column Chrom XP C18 150 × 0.30 mm, 3 mm particle size and 120 Å pore size (Eksigen, SCIEX), at a flow rate of 5 μL/min. Water and ACN, both containing 0.1% formic acid, were used as solvents A and B, respectively as before. The gradient run consisted of 5% to 95% B for 30 min, 90% B for 5 min, and finally 5% B for 5 min for column equilibration, for a total run time of 40 min. When the peptides were eluted, they were directly injected into a hybrid quadrupole-TOF mass spectrometer Triple TOF 6600 (SCIEX, Redwood City, CA, USA) operated with a DDA system in positive ion mode. A micro source (SCIEX) was used for the interface between microLC and MS, with an application of 2600 V voltage. The acquisition mode consisted of a 250 ms survey MS scan from 400 to 1250 m/z followed by an MS/MS scan from 100 to 1500 m/z (25 ms acquisition time) of the top 65 precursor ions from the survey scan, for a total cycle time of 2.8 s. The fragmented precursors were then added to a dynamic exclusion list for 15 s; any singly charged ions were excluded from the MS/MS analysis.

The peptide and protein identifications were performed as described before for DDA analysis using protein Pilot software Only peptides with a confidence score above 99% (as obtained from the Protein Pilot database search) were included in the spectral library.

**Relative quantification by SWATH-MS acquisition**

SWATH–MS acquisition was performed with a TripleTOF® 6600 LC-MS/MS system (SCIEX). Samples were analyzed using a data-independent acquisition (DIA) method (44 total samples). Each 4-μL sample (from a mg/ml solution) was analyzed with the LC-MS equipment and LC gradient described above for building the spectral library, but instead employing the SWATH-MS acquisition method. This method involved repeating a cycle that consisted of the acquisition of 100 TOF MS/MS scans (400 to 1500 m/z, high sensitivity mode, 50 ms acquisition time) of overlapping sequential precursor isolation windows of variable width (1 m/z overlap), covering the 400 to 1250 m/z mass range with a previous TOF MS scan (400 to 1500 m/z, 50 ms acquisition time) for each cycle. Total cycle time was 6.3 s. For each sample set, the width of the 100 variable windows was optimized according to the ion density found in the DDA runs using a SWATH-MS variable window calculator worksheet from SCIEX [7,8].

**SWATH-MS data analysis**

The targeted data extraction of the fragment ion chromatogram traces from the SWATH-MS runs was performed by PeakView (version 2.2) using the SWATH-MS Acquisition MicroApp (version 2.0). This application processed the data using the spectral library created from DDA analysis. Up to 10 peptides per protein and 7 fragments per peptide were selected, based on signal intensity; any shared and modified peptides were excluded from processing. Five-minute windows and 30-ppm widths were used to extract the ion chromatograms; SWATH-MS quantitation was attempted for all proteins in the ion library that were identified by ProteinPilot with an FDR below 1%. The retention times of the peptides were realigned in each run according to the internal retention time peptides corresponding to the various proteins identified in each sample and eluted along the whole-time axis. The peak areas for the peptides were obtained by summing the peak areas from the corresponding fragment ions. PeakView computed an FDR and a score for each assigned peptide according to the chromatographic and spectral components; only peptides with an FDR below 1% were used for protein quantitation.

The integrated peak areas (processed. mrkvw files from PeakView) were directly exported to MarkerView software (SCIEX) for relative quantitative analysis. The export generated 3 files containing quantitative information about individual ions, the total intensity of different ions for a particular peptide, and the total intensity of various peptides for a particular protein. MarkerView had been used for analysis of SWATH-MS data reported in other proteomics studies [7,8]. The software alignment of the data compensates for small variations in the mass and RT values, ensuring precise comparisons of identical compounds in different samples. To homogenise the data obtained, a most likely ratio (MLR) normalisation was performed [36].

The t-test indicates how well each variable distinguishes the 2 groups, reported as a p-value. For each library, its set of differentially expressed proteins (p-value <0.05) with a 2-fold increase or decrease was selected.
