# Supplementary material for: Infection-driven proteomic signatures in immune cell–derived extracellular vesicles reflect hemorrhagic stroke outcome
Source: J Neuroinflammation. 2025 Dec 4;23:22. doi: 10.1186/s12974-025-03635-9 (PMC12822078; doi:10.1186/s12974-025-03635-9)
Supplement: Supplementary file 2 — Supplementary Material 2: Table S1. Demographic data, risk factors, and clinical in patients with poor outcome with and without infection. Table S2. Proteins in T-cells, B-cells and monocytes-derived EVs in infected vs. non infected patients. Table S3. Statistical power (Cohen’s d and Hedges’ g) of selected proteins. Figure S1. Characterization of T-cells, B-cells and monocytes-derived EVs. Figure S2. Western blot for validation of the proteins found with differential abundance. [file 12974_2025_3635_MOESM2_ESM.docx]

**Supplementary material**

**Table S1. Demographic data, risk factors, and clinical in patients with poor outcome with and without infection.**

|  | **Poor outcome** | |  |
| --- | --- | --- | --- |
|  | **Infection** | **No infection** |  |
|  | **n=14** | **n=12** | ***p* value** |
| **Age, years, mean (SD)** | 72 (11.2) | 72 (9) | 0.899 |
| **Sex, male, *n* (%)** | 9 (64.3) | 7 (58.3) | 1 |
| **Hypertension, *n* (%)** | 12 (85.7) | 10 (83.3) | 1 |
| **Diabetes mellitus, *n* (%)** | 2 (14.3) | 3 (25.0) | 0.635 |
| **Dyslipidaemia, *n* (%)** | 10 (71.4) | 6 (50.0) | 0.422 |
| **Atrial fibrillation, *n* (%)** | 2 (14.3) | 4 (33.3) | 0.365 |
| **Baseline systolic blood pressure, mean (SD)** | 174.5 (23.2) | 171.9 (36.6) | 0.846 |
| **Baseline diastolic blood pressure, mean (SD)** | 93.1 (9.9) | 98.9 (30.9) | 0.519 |
| **Baseline glycemia, mean (SD)** | 127.9 (25.4) | 141.3 (45.3) | 0.705 |
| **Baseline O_2_ saturation, mean (SD)** | 95.5 (2.1) | 95.8 (2.8) | 0.778 |
| **Baseline NIHSS, median (IQR)** | 17.5 (11.3) | 19.5 (11.5) | 0.595 |
| **Baseline ICH volume mm^3^, mean (SD)** | 18.1 (10.5) | 17.3 (14.2) | 0.875 |
| **7d NIHSS, median (IQR)** | 18 (12) | 11(8) | 0.186 |
| **7d ICH volume mm^3^, mean (SD)** | 19.2 (11.8) | 17.3 (16.2) | 0.501 |
| **3mo NIHSS, median (IQR)** | 4 (17) | 9 (10.5) | 0.824 |
| **3mo mRS, median (IQR)** | 3 (1) | 4 (1.3) | 0.257 |
| **6mo NIHSS, median (IQR)** | 6 (11) | 7 (6.5) | 0.549 |
| **6mo mRS, median (IQR)** | 3 (1.5) | 4 (1) | 0.776 |

Abbreviations: IQR: interquartile range; mRS: modified Rankin Scale; NIHSS: National Institutes of Health Stroke Scale score; SD: standard deviation.

**Table S2.** **Proteins in T-cells, B-cells and monocytes-derived EVs in infected vs. non infected patients.** Proteins with higher abundance are marked in green, and those with lower abundance in red. For detailed data with fold change and p-value see “Supplementary material proteomic.xlsx” file which contain the RAW data of the proteomic study.

| T-cells-derived EVs |  | B-cells-derived EVs |  | Monocytes-derived EVs |
| --- | --- | --- | --- | --- |
| Q8NBP7\|PCSK9 |  | P31040\|SDHA |  | P01011\|AACT |
| Q06323\|PSME1 |  | Q12905\|ILF2 |  | Q93084\|AT2A3 |
| P09936\|UCHL1 |  | P31948\|STIP1 |  | Q12904\|AIMP1 |
| Q99873\|ANM1 |  | P13671\|CO6 |  | Q99808\|S29A1 |
| Q13630\|FCL |  | Q9Y230\|RUVB2 |  | P05556\|ITB1 |
| O15144\|ARPC2 |  | P01857\|IGHG1 |  | Q9UQ80\|PA2G4 |
| P42285\|MTREX |  | P62249\|RS16 |  | P40926\|MDHM |
| P13798\|ACPH |  | Q9NVD7\|PARVA |  | Q9NY33\|DPP3 |
| P62807\|H2B1C |  | P04216\|THY1 |  | Q15691\|MARE1 |
| P15586\|GNS |  | O00303\|EIF3F |  | P30405\|PPIF |
| P14780\|MMP9 |  | P25786\|PSA1 |  | P43007\|SATT |
| Q13085\|ACACA |  | P25205\|MCM3 |  | Q96AY3\|FKB10 |
| P00352\|AL1A1 |  | P08758\|ANXA5 |  | Q9UM07\|PADI4 |
| Q13813\|SPTN1 |  |  |  | P80404\|GABT |
| P15311\|EZRI |  | Q00796\|DHSO |  | P00167\|CYB5 |
|  |  | P27797\|CALR |  | P07202\|PERT |
| P80188\|NGAL |  | P50995\|ANX11 |  | P14314\|GLU2B |
| P00367\|DHE3 |  | P41252\|SYIC |  |  |
| P23142\|FBLN1 |  | P06396\|GELS |  | P19823\|ITIH2 |
| O14974\|MYPT1 |  | P00751\|CFAB |  | Q9BW30\|TPPP3 |
| Q96FJ2\|DYL2 |  | P34741\|SDC2 |  | P23381\|SYWC |
| P30046\|DOPD |  | Q5SSJ5\|HP1B3 |  | O75964\|ATP5L |
|  |  | A0A0C4DH31\|HV118 |  | Q8NBP7\|PCSK9 |
|  |  | Q02809\|PLOD1 |  | P28838\|AMPL |
|  |  | Q00013\|EM55 |  | P61981\|1433G |
|  |  | P31937\|3HIDH |  | P15169\|CBPN |
|  |  | P17655\|CAN2 |  | P62195\|PRS8 |
|  |  | Q9UMD9\|COHA1 |  | P00742\|FA10 |
|  |  | Q9BT78\|CSN4 |  | P31327\|CPSM |
|  |  | P08575\|PTPRC |  | P0C0L4\|CO4A |
|  |  | P17931\|LEG3 |  | Q99714\|HCD2 |
|  |  | P28482\|MK01 |  | P11169\|GTR3 |
|  |  | P02787\|TRFE |  | Q9UBW5\|BIN2 |
|  |  | P63279\|UBC9 |  | Q02094\|RHAG |
|  |  | P61604\|CH10 |  | P02765\|FETUA |
|  |  | Q13148\|TADBP |  | P18428\|LBP |
|  |  | Q8N163\|CCAR2 |  | Q70J99\|UN13D |
|  |  | Q9NP58\|ABCB6 |  | P04217\|A1BG |
|  |  | P00738\|HPT |  | O14974\|MYPT1 |
|  |  | P83731\|RL24 |  | Q96QK1\|VPS35 |
|  |  |  |  | Q9UNW1\|MINP1 |
|  |  |  |  | Q9NTK5\|OLA1 |
|  |  |  |  | Q9Y608\|LRRF2 |
|  |  |  |  | P28482\|MK01 |
|  |  |  |  | Q9UK55\|ZPI |
|  |  |  |  | P04216\|THY1 |
|  |  |  |  | P02741\|CRP |
|  |  |  |  | O96000\|NDUBA |
|  |  |  |  | O75594\|PGRP1 |
|  |  |  |  | O75746\|CMC1 |

**Table S3. Statistical power (Cohen’s d and Hedges’ g) of selected proteins.**

| **Protein** | **Cohen’s d** | **Hedges’ g** | **SD of g** | **Lower Confidence Limit** | **Upper Confidence Limit** | **Power** |
| --- | --- | --- | --- | --- | --- | --- |
| **COHA1** | -7.635 | -6.108 | 1.847 | -9.728 | -2.488 | 1.000 |
| **H2B1C** | 6.677 | 5.342 | 1.646 | 2.116 | 8.568 | 1.000 |
| **MTREX** | 6.201 | 4.961 | 1.548 | 1.927 | 7.995 | 1.000 |
| **PSME1** | 5.020 | 4.016 | 1.310 | 1.448 | 6.584 | 0.993 |
| **PCASK9** | -4.965 | -3.972 | 1.300 | -6.519 | -1.425 | 0.992 |
| **CMC1** | NA | NA | NA | NA | NA | NA |

Abbreviations: SD: standard deviation.


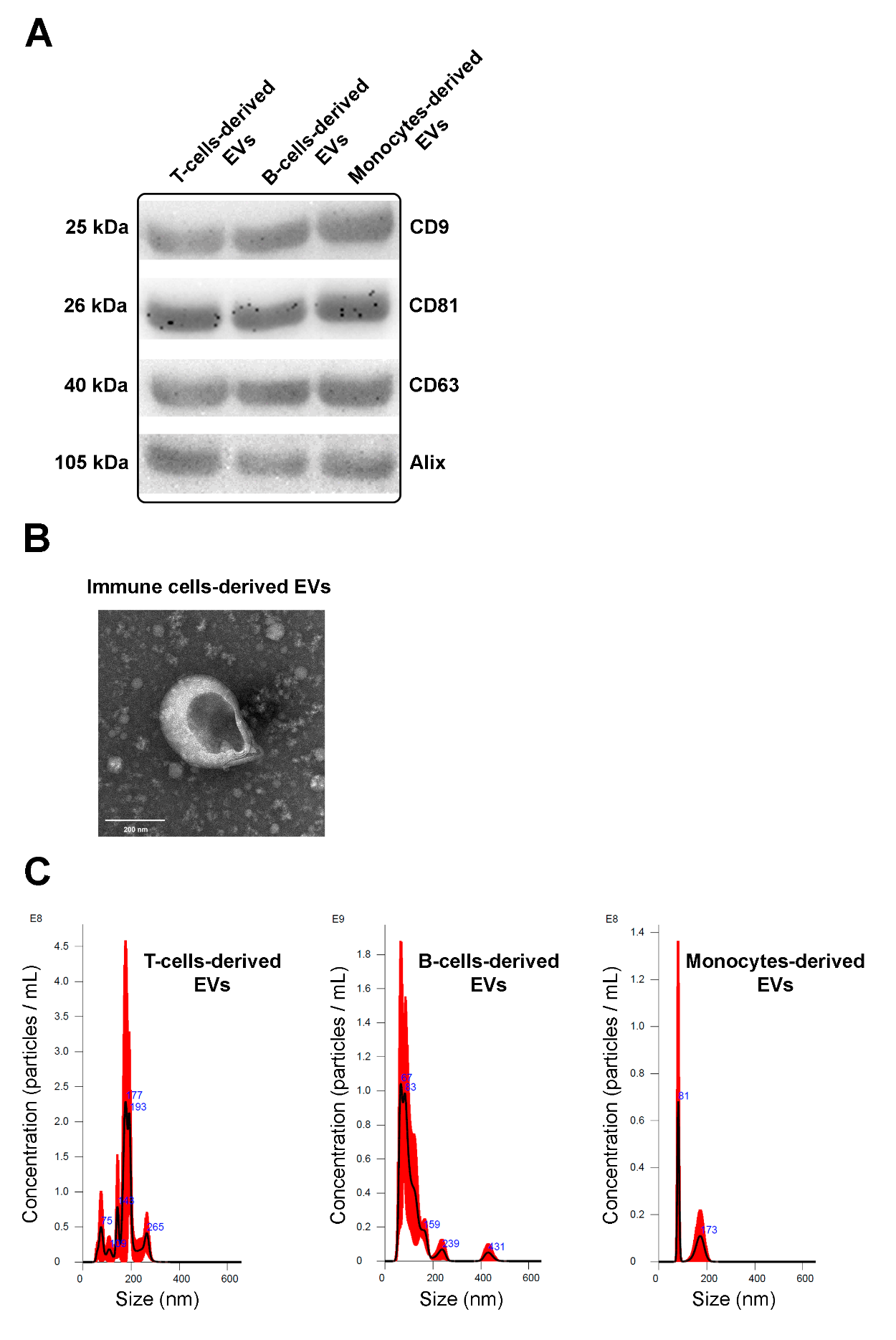


**Figure S1. Characterization of T-cells, B-cells and monocytes-derived EVs. (A)** Presence of CD9, CD81, CD63 and Alix as specific markers of immune cell-derived EVs. **(B)** Size and shape of immune cells-derived EVs by transmission electron microscopy imaging (Scale bar 200nm). **(C)** Size and concentration of immune cell-derived EVs by nanoparticle tracking analysis. Abbreviations: EVs: extracellular vesicles.


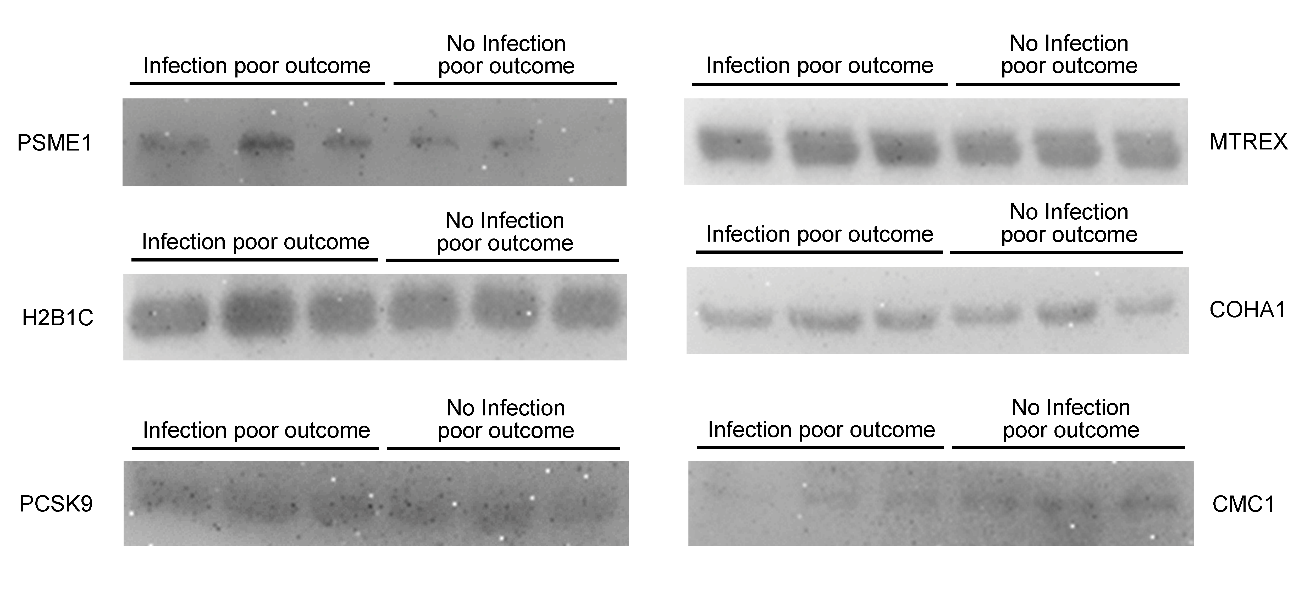


**Figure S2. Western blot for validation of the proteins found with differential abundance.**
