# Supplementary material for: Infection-driven proteomic signatures in immune cell–derived extracellular vesicles reflect hemorrhagic stroke outcome
Source: J Neuroinflammation. 2025 Dec 4;23:22. doi: 10.1186/s12974-025-03635-9 (PMC12822078; doi:10.1186/s12974-025-03635-9)
Supplement: Supplementary file 4 — Supplementary Material 4. [file 12974_2025_3635_MOESM4_ESM.pptx]

## Slide 1
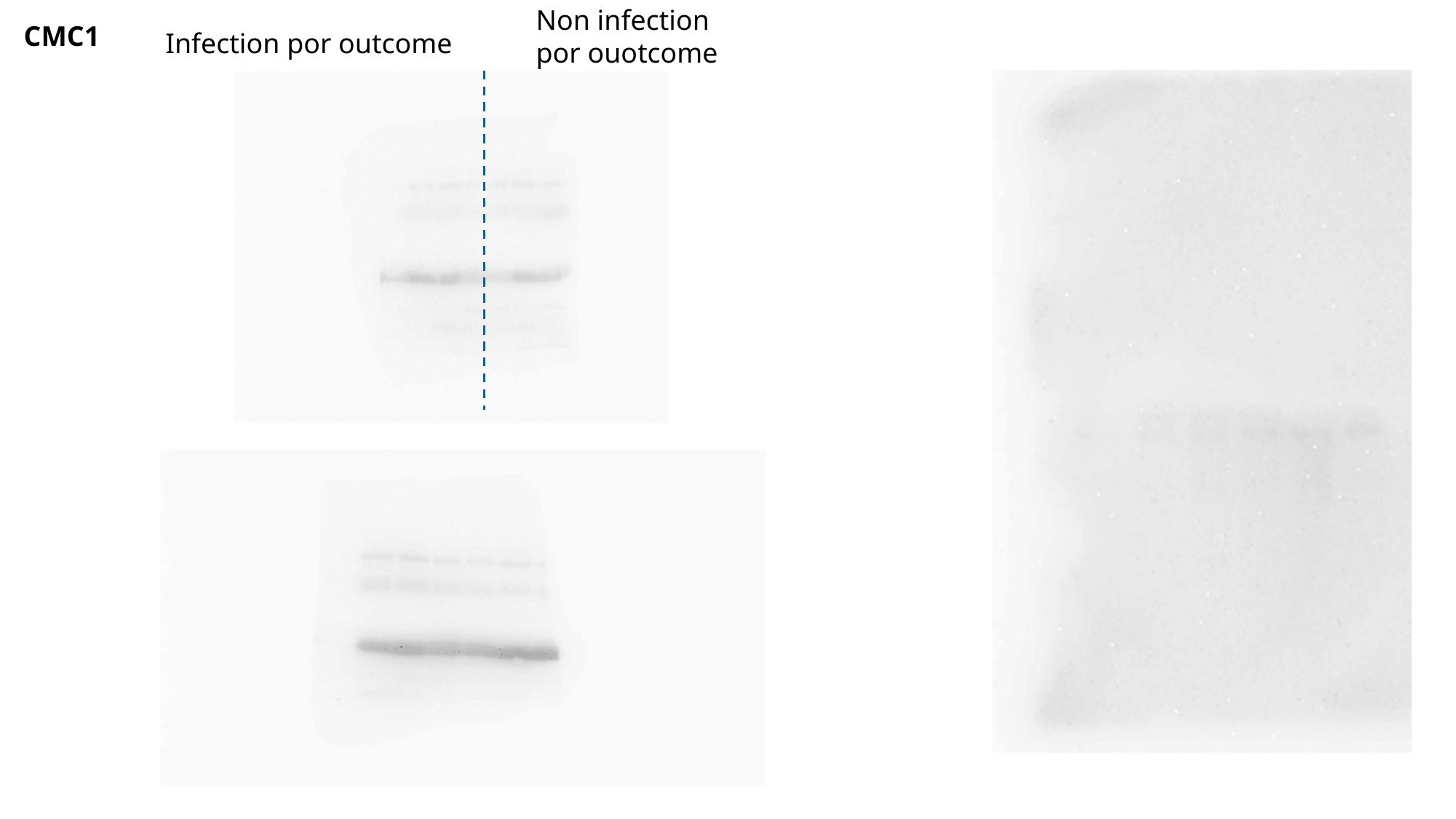

Non infection
por ouotcome
CMC1
Infection por outcome

## Slide 2
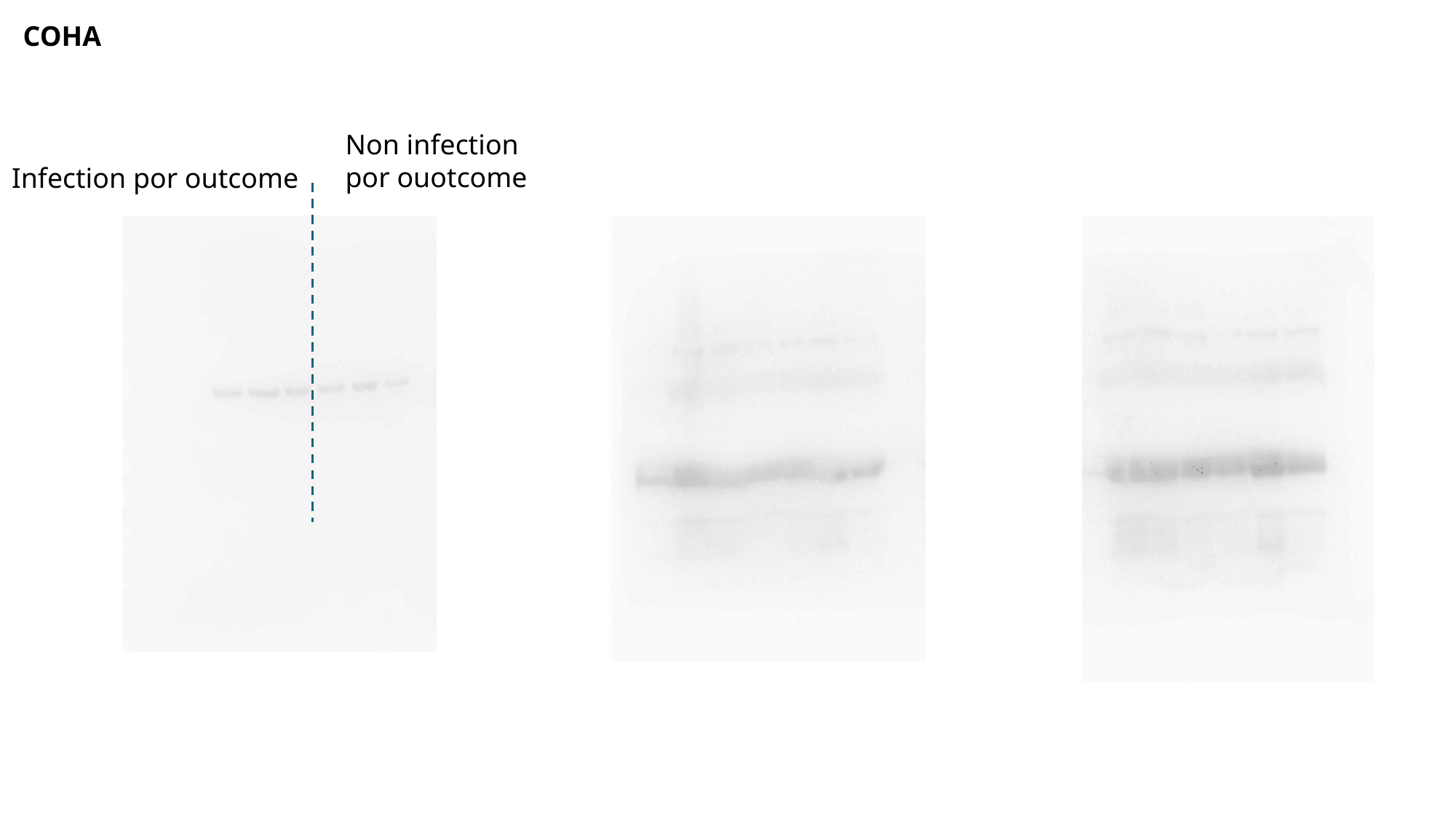

COHA
Non infection
por ouotcome
Infection por outcome

## Slide 3
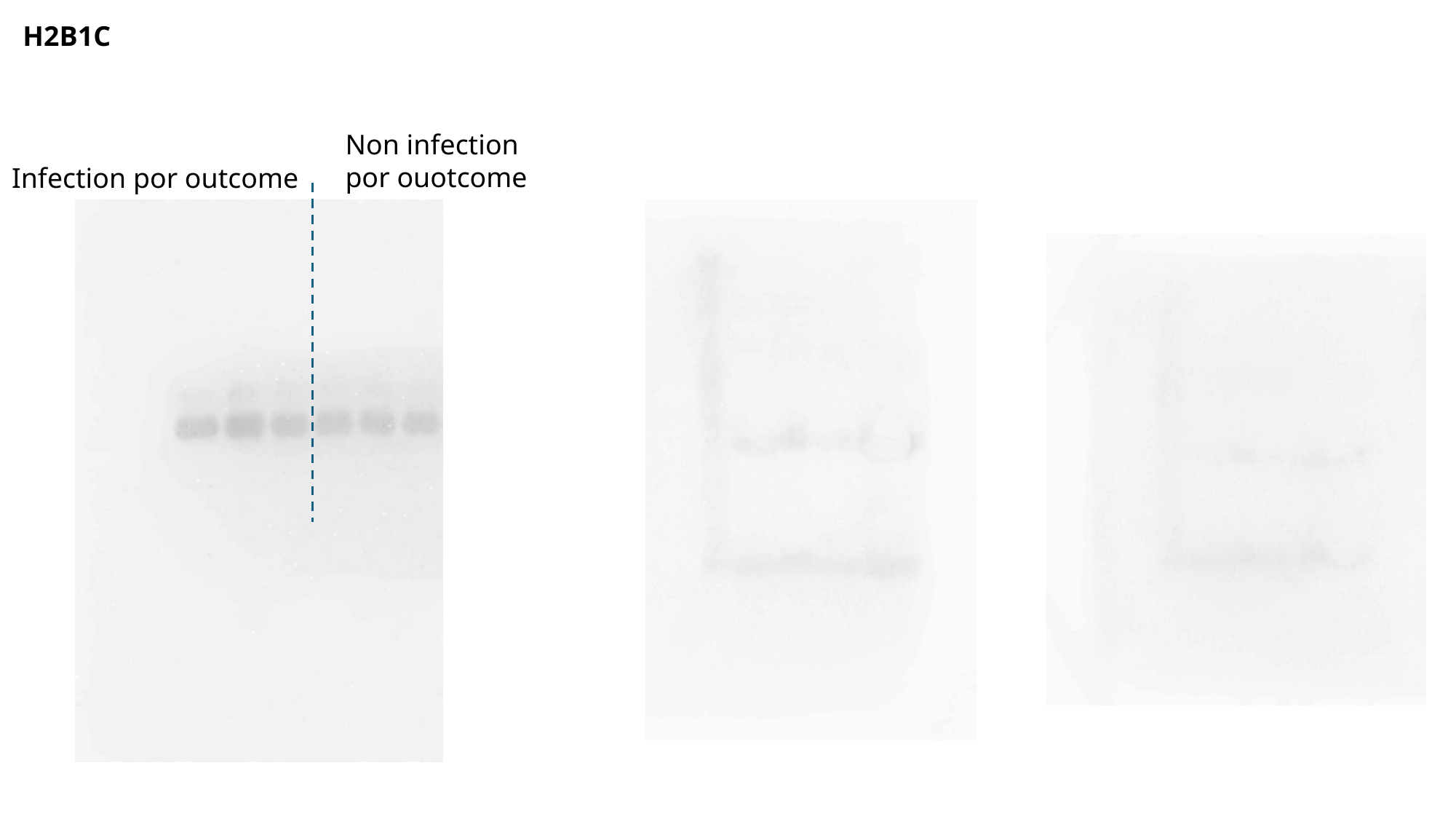

H2B1C
Non infection
por ouotcome
Infection por outcome

## Slide 4
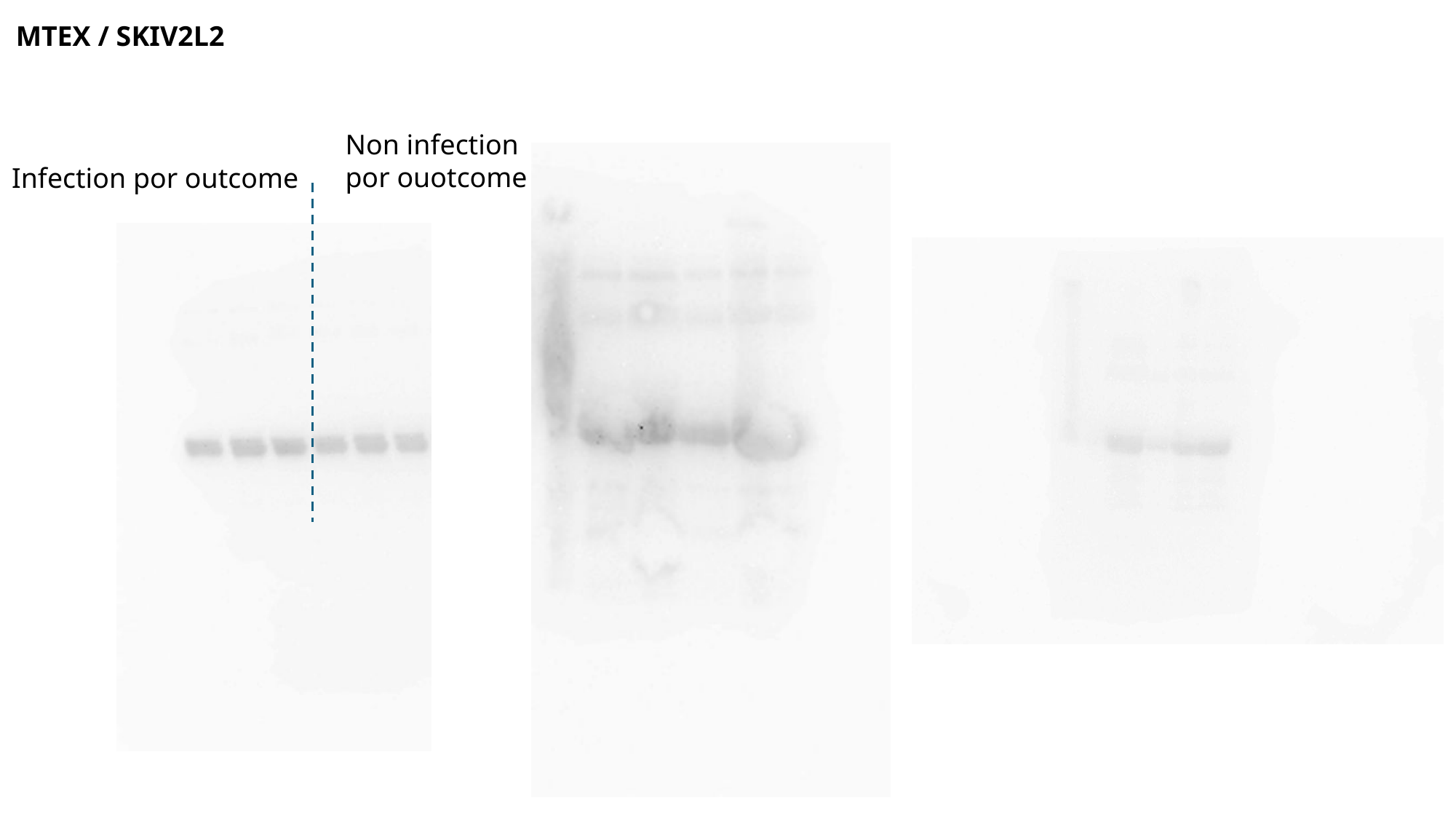

MTEX / SKIV2L2
Non infection
por ouotcome
Infection por outcome

## Slide 5
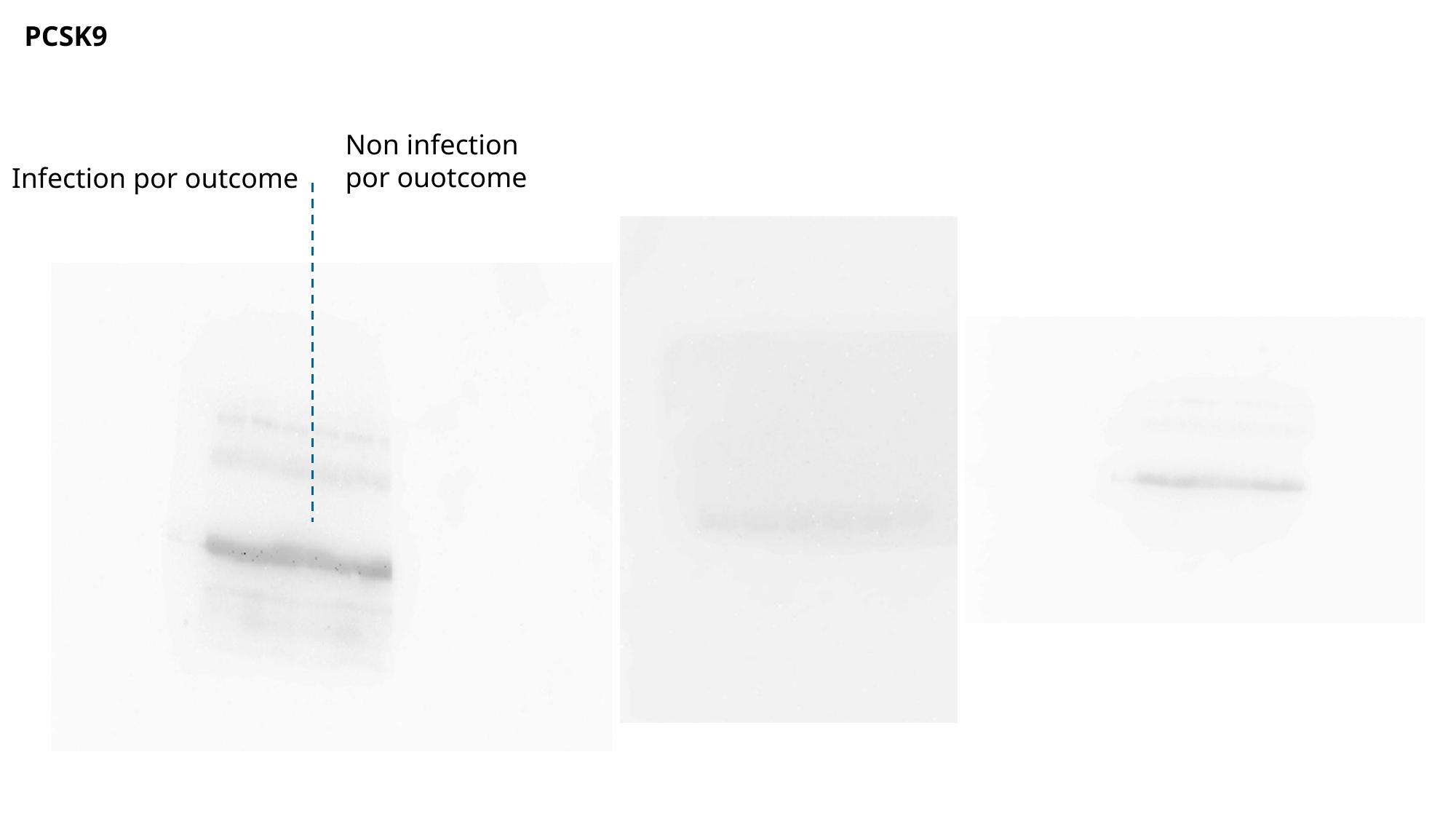

PCSK9
Non infection
por ouotcome
Infection por outcome

## Slide 6
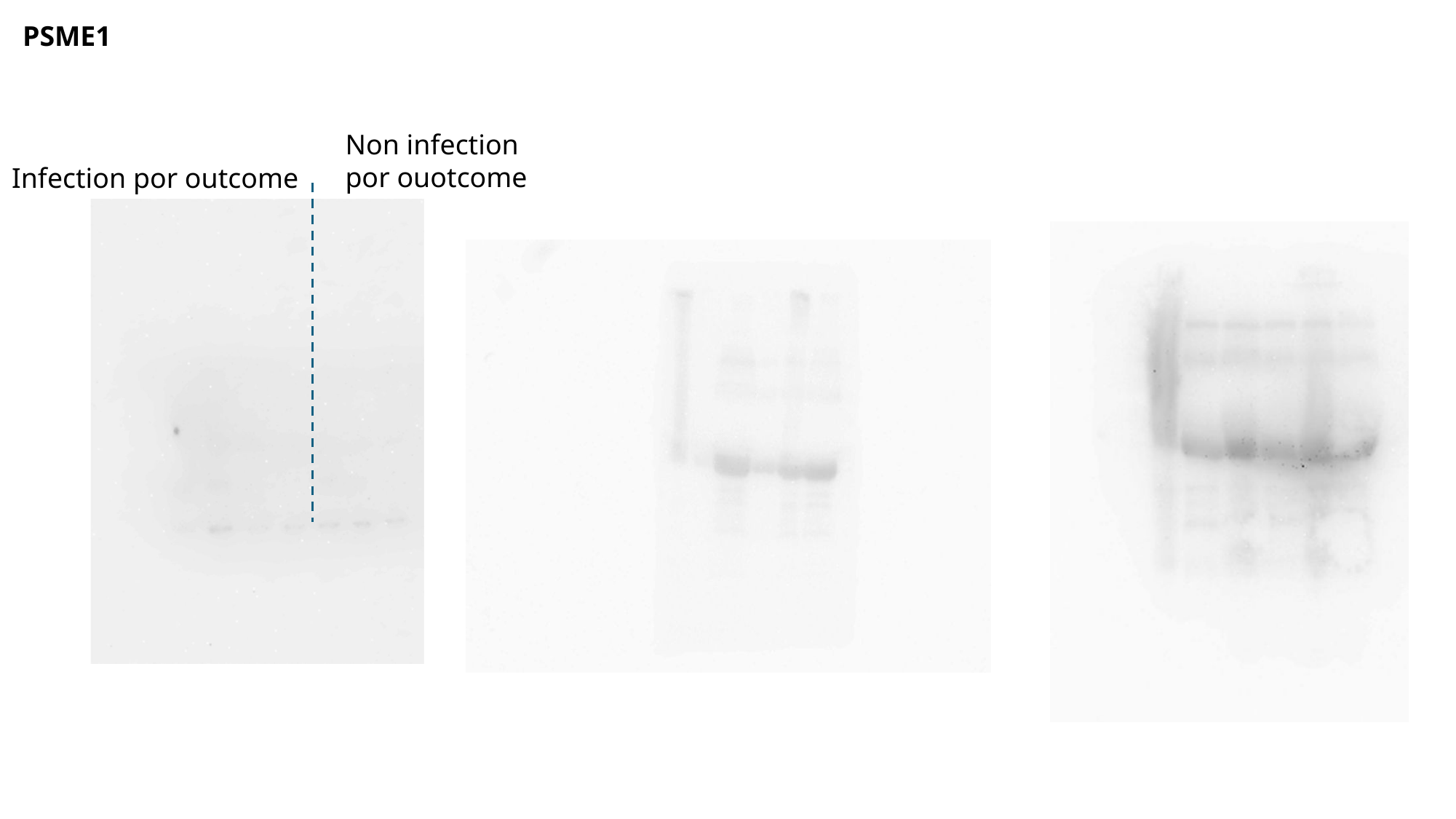

PSME1
Non infection
por ouotcome
Infection por outcome
